# PATTERNS OF ALCOHOL CONSUMPTION AND MENTAL HEALTH IN HCV-INFECTED PATIENTS: IMPLICATIONS FOR ANTIVIRAL ELIGIBILITY

**DOI:** 10.1590/S0004-2803.24612025-054

**Published:** 2025-12-01

**Authors:** Lívia Beraldo de LIMA, André MALBERGIER, Priscila Dib GONÇALVES, Suzane Kioko ONO, Anderson Sousa MARTINS-DA-SILVA, Mário Guimarães PESSOA, Mariana Akemi NABESHIMA, Maria Cassia MENDES-CORRÊA, Arthur Guerra de ANDRADE, João Maurício CASTALDELLI-MAIA

**Affiliations:** 1 University of São Paulo, University of São Paulo School of Medicine, Department of Psychiatry, São Paulo, SP, Brazil.; 2 Columbia University, Department of Psychiatry, New York, NY, USA.; 3 University of São Paulo, University of São Paulo School of Medicine, Department of Gastroenterology and Hepatology, São Paulo, SP, Brazil.; 4 University of São Paulo, University of São Paulo School of Medicine, Laboratory of Medical Research in Virology (LIM 52), São Paulo, SP, Brazil.

**Keywords:** Hepatitis C, alcohol drinking, depression, direct-acting antivirals, comorbidity, Hepatite C, consumo de bebidas alcoólicas, depressão, antivirais, comorbidade

## Abstract

**Background:**

Hepatitis C virus (HCV) infection remains a significant public health concern. Behavioral factors such as alcohol use and psychiatric comorbidities, particularly depression, may influence disease progression and eligibility for antiviral treatment.

**Objective:**

To assess the prevalence and clinical impact of current alcohol consumption in patients with chronic HCV infection eligible for treatment with direct-acting antivirals (DAAs), and to explore its associations with depressive symptoms and clinical characteristics.

**Methods:**

This was a cross-sectional study including 109 monoinfected HCV outpatients. Sociodemographic, clinical, and laboratory data were collected. Current alcohol use was assessed using the alcohol, smoking and substance involvement screening test (ASSIST), and depressive symptoms were measured by the Beck Depression Inventory (BDI).

**Results:**

Among participants, 46 (43.4%) reported current alcohol consumption. No statistically significant difference in alcohol use was found between male and female participants (*P*=0.15). Women showed significantly higher depressive symptom scores compared to men (11.33 vs 7.67; *P*=0.024). However, no significant association was observed between levels of alcohol consumption (<12 g/day, ≥12 g/day, or abstinent) and the presence of moderate to severe depressive symptoms. Additionally, alcohol use was positively associated with tobacco use (*P*=0.01), but not with clinical comorbidities.

**Conclusion:**

Moderate alcohol use in HCV-infected patients was not associated with increased depressive symptoms or worse clinical parameters prior to DAA therapy. These findings suggest that moderate alcohol consumption should not be an absolute contraindication for initiating HCV treatment, though further studies are needed.

## HIGHLIGHTS

• Alcohol use and psychiatric comorbidities may influence hepatitis C virus (HCV) progression and eligibility for antiviral treatment.

• This was a cross-sectional study that included 109 monoinfected HCV outpatients.

• Moderate alcohol use in HCV-infected patients was not associated with increased depressive symptoms or worse clinical parameters.

•Moderate alcohol consumption should not be an absolute contraindication for initiating HCV treatment.

## RESUMO

**Contexto:**

A infeção pelo vírus da hepatite C (VHC) continua a ser um importante problema de saúde pública. Os fatores comportamentais, como o consumo de álcool e as comorbilidades psiquiátricas, em particular a depressão, podem influenciar a progressão da doença e a elegibilidade para o tratamento antiviral.

**Objetivo:**

Avaliar a prevalência e o impacto clínico do consumo atual de álcool em doentes com infecção crônica pelo VHC elegíveis para tratamento com antivirais de ação direta (AAD), e explorar as suas associações com sintomas depressivos e caraterísticas clínicas.

**Métodos:**

Este foi um estudo transversal que incluiu 109 doentes monoinfectados com VHC em ambulatório. Foram recolhidos dados sociodemográficos, clínicos e laboratoriais. O consumo atual de álcool foi avaliado através do alcohol, smoking and substance involvement screening test (ASSIST), e os sintomas depressivos foram medidos através do inventário de depressão de beck (BDI).

**Resultados:**

Entre os participantes, 46 (43,4%) relataram consumo atual de álcool. Não foi encontrada uma diferença estatisticamente significativa no consumo de álcool entre os participantes do sexo masculino e feminino (*P*=0,15). As mulheres apresentaram pontuações de sintomas depressivos significativamente mais elevadas em comparação com os homens (11,33 vs 7,67; *P*=0,024). No entanto, não foi observada associação significativa entre os níveis de consumo de álcool (<12 g/dia, ≥12 g/dia ou abstinente) e a presença de sintomas depressivos moderados a graves. Adicionalmente, o consumo de álcool foi positivamente associado ao consumo de tabaco (*P*=0,01), mas não às comorbilidades clínicas.

**Conclusão:**

O consumo moderado de álcool em doentes infectados com VHC não foi associado a um aumento dos sintomas depressivos ou a piores parâmetros clínicos antes da terapêutica com AAD. Estes resultados sugerem que o consumo moderado de álcool não deve ser uma contraindicação absoluta para iniciar o tratamento do VHC, embora sejam necessários mais estudos.

## INTRODUCTION

Hepatitis C remains a major global public health challenge, with considerable social and economic burdens. It is estimated that approximately 1% of the global population (~71 million people) is infected with the hepatitis C virus (HCV), resulting in about 365,000 deaths annually due to its complications, such as liver cirrhosis and hepatocellular carcinoma (HCC)^1^. HCC alone accounts for roughly 260,000 new cases each year. In response, the World Health Organization (WHO) launched a global strategy in 2016 to eliminate HCV as a public health threat by 2030, setting goals of an 80% reduction in new infections and a 65% reduction in HCV-related deaths^2^.

Although much is known about the virology and pathophysiology of HCV, behavioral and psychiatric factors that influence disease progression and treatment eligibility remain underexplored, particularly in populations without severe substance use disorders. Alcohol consumption is a well-established co-factor that accelerates liver fibrosis in individuals with HCV and is associated with poorer immune responses, increased oxidative stress, and greater hepatotoxicity^5^ ^,^ ^7^ ^,^ ^8^. Despite this, many individuals with chronic HCV continue to consume alcohol at varying levels of intensity, and data on moderate users are scarce. In clinical practice, this lack of evidence has contributed to inconsistent approaches regarding the eligibility of non-abstinent patients for antiviral therapy.

Gender differences are also notable in the context of HCV. Men are twice as likely to develop chronic HCV infection^6^, whereas women tend to exhibit slower fibrotic progression and more favorable liver biochemistry profiles^3^. These differences have been partly attributed to the anti-fibrotic role of estrogen^4^. However, the intersection between gender, alcohol consumption, and mental health symptoms - such as depression - in HCV - infected populations remains poorly characterized.

There is also limited evidence regarding the association between current alcohol use and psychiatric symptoms - such as depression - which are common among HCV - infected individuals and may impact both quality of life and treatment adherence. A French study by Schwarzinger and colleagues^10^ highlighted alcohol use disorders as one of the leading causes of disease progression, liver transplantation, and premature death in people with chronic HCV, yet most studies focus only on those meeting criteria for addiction, excluding moderate or occasional users.

Given these gaps, this study aimed to: (1) describe the frequency and patterns of current alcohol consumption in a sample of HCV-infected outpatients eligible for treatment with direct-acting antivirals (DAAs); (2) examine gender differences in depressive symptoms and alcohol use; and (3) explore associations between current alcohol use, depressive symptoms, and clinical comorbidities. These findings may help inform more nuanced, evidence-based approaches to managing patients with HCV who do not meet criteria for alcohol dependence but still engage in potentially harmful drinking behavior.

## METHODS

### Study population and setting

This is a cross-sectional study that evaluated patients with chronic HCV infection who were candidates for treatment with direct-acting antivirals (DAAs). Data was collected from April 2017 to July 2018. Subjects were outpatients at the gastroenterology and hepatology department and at the infectious and parasitic diseases department of the Clinical Hospital, University of Sao Paulo Medical School.

The following inclusion criteria were used in this study: subjects over 18 years old; both genders; patients with HCV mono-infection eligible for the therapeutic regimen with DAAs. We excluded patients co-infected with hepatitis B virus (HBV) and/or Acquired Immunodeficiency Virus (HIV); patients with other mental disorders (such as schizophrenia, bipolar affective disorder, major depression, dementia, alcohol dependence); history of severe head injury; patients treated with interferon; patients submitted to liver transplantation.

The study was approved by the institutional Research Ethics Committee with certificate of ethical presentation and appreciation number 65133315.7.0000.0068. After being informed about the study objectives, all individuals who agreed to participate signed an informed consent form.

### Instruments

A standardized sociodemographic and clinical form was applied to collect information concerning the patient’s age; gender; education level; occupation; marital status; current and history of alcohol and other substances use; major psychiatric symptoms; treatments already performed; and family history. Data was obtained by an interpersonal interview conducted by the first author (L.B.L., clinical psychiatrist) at the hospital outpatient service where all assessment instruments were applied.

Structured clinical interview for DSM-IV (SCID) is a semi-structured interview developed to perform the major axis I diagnose based on DSM-IV (diagnostic and statistical manual of mental disorders)^11^. In the SCID, alcohol use is evaluated in the E module (substance use disorder), the various patterns of consumption are investigated e.g., lifetime and current use, and dependence^9^.

Alcohol, smoking and substance screening test (ASSIST) was developed by the World Health Organization (WHO), translated, and validated in Brazil by Henrique et al. 2004^12^. This is a structured questionnaire containing eight questions about the use of nine classes of psychoactive substances (tobacco, alcohol, marijuana, cocaine, stimulants, sedatives, inhalants, hallucinogens, and opiates). The questions address the overall frequency of usage, the frequency of usage in the last three months, problems related to usage, concern about usage by third parties, impairment in the performance of expected tasks, unsuccessful attempts to interrupt or reduce usage, feeling of compulsion and usage of injectable substances. Each response corresponds to a score from 0 to 4, and the total can vary from 0 to 20. Score 0 to 3 is considered an indicative of occasional use; 4 to 15, abusive use and 16, dependence^13^. ASSIST was considered positive for alcohol use in this study when scored positive on question two and negative on other issues.

Beck Scale is an inventory used as a measure of self-evaluation of depression validated for the Brazilian population. The Beck Scale is composed by the BAI (Beck Anxiety Inventory) and the BDI (Beck Depression Inventory). In this research, the BDI was used to evaluate depressive symptoms. BDI was originally developed by Beck et al. 1961^14^. It is a self-report scale for the assessment of the intensity of depressive symptoms composed of 21 items each one with four alternatives, in ascending order, for the level of depression. Patients are instructed to choose the answer that best suits their previous week. The sum of the scores identifies the level of depression. The severity of depression is as follows: minimum (from 0 to 11 points); light (12 to 19 points); moderate (from 20 to 35 points); and severe (from 36 to 63 points). In Brazil, the BDI was translated and validated by Andrade et al. 2001^15^.

### Laboratorial data

Subjects were assessed by mean corpuscular volume (MCV), hemoglobin (Hb), hematocrit (Ht), gamma glutamyl transferase (GGT), alkaline phosphatase, urea, creatinine, alanine aminotransferase (ALT), aspartate aminotransferase (AST), sodium (Na), potassium (K), viral genotyping and HCV quantification by polymerase chain reaction (PCR).

### Statistical analysis

The data was analyzed using SPSS software version 24. The continuous and non-continuous data of the variables was compared with the Gaussian curve and determined as parametric or non-parametric using the KS (Kolmogorov-Smirnov) distance test and the Shapiro-Wilks test. It was represented by mean and standard deviation (parametric data) or by median and percentile 25 to 75 (non-parametric data).

For parametric data, when comparing between two independent groups, the student’s t-test with or without the welch correction was used, depending on the performance in the Levene Test. When comparing three or more independent groups with similar variances (according to Bartlet’s test), the variance analysis test was used for non-repeated measurements with tukey’s post-test for groups with similar coefficient of variation. Still for non-repeated measurements, for groups with different coefficients of variation, the test of minimum significant difference (modified tukey test) was used. In the two-time comparison the paired student’s t-test was used. When three or more times were compared, the test used was the analysis of variance for repeated measures with post-test of minimum significant difference.

For non-parametric data analysis, when comparing two independent groups, the mann-whitney test with the bonferroni correction was used. When comparing three or more groups the kruskal-wallis test was used with the bonferroni correction and the post-test of dwass-steel-critchlow-flinger.

Categorical data was represented by absolute (n) and relative (%) frequency and the contingency matrices were analyzed by pearson’s chi-square test or fisher’s exact test when necessary. Complex matrices were partitioned into simple matrices for better determination of causality. A 5% Risk for Type I Error and 20% Risk for Type II Error was considered for the present study.

## RESULTS

### Demographics and clinical characteristics

From a total of 250 eligible patients, 130 did not have adequate medical records and five did not attend the initial interview. Of the 115 patients who attended the initial interview, two refused and 113 agreed to participate. Four were excluded: one who had a previous diagnosis of schizophrenia, two who were diagnosed with bipolar affective disorder and one who was deceased before the evaluation. Thus, 109 patients met the eligibility criteria and were included.

Table 1 shows that sixty (59%) of the participants were men, mostly married (59%), over 60 years of age (44%) and had elementary school educational level (53%). Most patients in this study (61%) showed liver fibrosis stages between F3 and F4 which represents greater impact in liver fibrosis.

**TABLE 1:** Sociodemographic characterization of the total sample (n=109 patients) stratified by gender.

	Male	Female	Total
	n (%)	n (%)	n (%)
Number of participants (%)	60 (59.0)	49 (41.0)	109 (100)
Age range (years)			
<39 years	5 (8.3)	3 (6.1)	8 (7.0)
40-49 years	15 (25.0)	10 (20.4)	25 (23.0)
50-59 years	13 (21.7)	14 (28.6)	27 (25.0)
60-69 years	19 (31.7)	19 (38.8)	38 (35.0)
70-79 years	8 (13.3)	3 (6.1)	11 (10.0)
Marital status			
Marriage	42 (70.0)	23 (47.0)	65 (60.0)
Divorced	5 (8.3)	9 (18.4)	14 (13.0)
Single	11 (18.4)	8 (16.2)	19 (17.0)
Widowed	2 (3.3)	9 (18.4)	11 (10.0)
Complete elementary school			
No	24 (40.0)	24 (49.0)	48 (47.0)
Yes	36 (60.0)	25 (51.0)	61 (53.0)

Table 2 shows the comparison of laboratorial findings according to gender. The only clinical variable with statistically significant difference was hemoglobin (*P*=0.02).

**TABLE 2:** Comparison of laboratory analysis data according to gender.

Exame	Male			Female		
	Reference	Mean	±SD	Reference	Mean	±SD
ALT (U/L)	<41	35.0	±	<31	31.0	±
AST (U/L)	<37	32.83	±	<31	30.33	±
ALP (U/L)	40-129	81.50	±	35-104	83.33	±
GGT (U/L)	8-61	47.83	±	5-36	27.33	±
TBIL (mg/dL)	0.2-1.0	1.15	±	0.2-1.0	1.09	±
DBIL (mg/dL)	0.1-0.3	0.68	±	0.1-0.3	0.63	±
IBIL (mg/dL)	0.2-0.7	0.47	±	0.2-0.7	0.49	±
Creatinine (mg/dL)	0.7-1.4	1.87	±	0.7-1.4	1.90	±
Plateletes (mm^3^)	140.000-400.000	159.000	±	140.000-450.000	190.000	±
Hemoglobin (g/dL)	13.5-17.5	14.03	±	12.0-16.0	12.83	±
Hematocrit (%)	41-53	41.03	±	36-46	39.13	±
MCV (fL)	80-100	90.06	±	80-100	90.00	±
Sodium (mmol/dL)	135-145	139.60	±	135-145	139.59	±
Urea (mg/dL)	20-40	43.71	±	20-40	43.91	±
Potassium (mmol/dL)	3-5.5	4.54	±	3-5.5	4.54	±
Albumin (g/dL)	3.5-5.2	4.45	±	3.4-4.9	4.33	±

ALP: alkaline phosphatase; ALT: alanine transaminase; AST: aspartate aminotransferase; DBIL: direct bilirubin; GGT: gamma-glutamyl transferase; IBIL: indirect bilirubin; MCV: mean corpuscular volume; TBIL: total bilirubin. *Mann Whitney test.

### Alcohol usage characteristics (ASSIST test)

From a total of 109 patients interviewed 46 (43.4%) rated positive for current alcohol usage and 63 (56.6%) had not used alcohol for at least three months. Considering the patients with positive ASSIST for alcohol use, 9 (8.25%) had used 1-2 times in the last three months, 18 (16.51%) had used weekly and 19 (17.43%) had used monthly.

Considering the results of the ASSIST questionnaire, Table 3 shows that 57.8% did not score positive for alcohol usage, of which 50.8% were female. Among those who tested positive for alcohol usage, 63.1% were male. There was no statistically significant difference between two groups (*P*=0.15).

**TABLE 3:** Comparison between the male and female genders in relation to negative (n=63) and positive (n=46) ASSIST.

ASSIST	Positive n (%)	Negative n (%)	*P
Female	17 (36.9)	32 (50.8)	0.15
Male	29 (63.1)	31 (49.2)	

ASSIST: alcohol, smoking and substance screening test. *chi-square test.

### Characterization of the depressive symptoms

Regarding depressive symptoms, stratifying the data according to gender (Table 4), the female group had a higher score (average 11.33) than the male group (average 7.67). There was a statistically significant difference (*P*=0.024).

**TABLE 4:** Characterization of depressive symptoms according to gender.

	Male n=60		Female n=49		*P
	Mean	±SD	Mean	±SD	
BDI	7.67	±8.00	11.33	±8.07	0.024

BDI: depression inventor, SD: standard deviation. *Mann Whitney test.

### Characterization of stratified sample according to alcohol use

Table 5 shows the results of the comparison of sociodemographic data between positive and negative ASSIST test. No statistically significant differences were observed when comparing age, gender, marital status and HCV genotype.

**TABLE 5:** Comparison of the sociodemographic data between positive and negative ASSIST test.

	Positive n (%)	Negative n (%)	*P
Age range (years)			
<39	5 (10.86)	3 (4.76)	0.69
40 a 59	12 (26.08)	13 (20.63)	
50 a 59	11 (23.93)	17 (27.00)	
60 a 69	14 (30.45)	24 (38.09)	
70 a 79	4 (8.69)	6 (6.34)	
Gender			
Female	17 (36.95)	32 (50.80)	0.15
Male	29 (63.05)	31 (49.20)	
Marital status			
Married	27 (58.70)	38 (60.31)	0.96
Divorced/widowed	11 (23.9)	13 (20.64)	
Single	8 (17.40)	12 (19.05)	

ASSIST: alcohol, smoking and substance screening test. *chi-square test.

In the positive ASSIST test group, a higher prevalence of smokers was observed (68.4% vs 31.6%, *P*=0.01). The presence of clinical comorbidities was higher in the negative ASSIST test group (hypertension: 67.3% vs 32.7% *P*=0.04; hypothyroidism: 92.9% vs 7.1% *P*=0.043; chronic kidney disease: 88.9% vs 11.1%, *P*=0.003).

### Association between alcohol usage and depressive symptoms

When comparing reported depressive symptoms from moderate to severe BDI with daily alcohol consumption (<12 g and >12 g alcohol/day), no statistically significant differences were observed between the analyzed groups (Table 6).

**TABLE 6:** Association between alcohol use and depressive symptoms according to alcohol consumption.

	ASSIST negative	<12 g alcohol/day	≥12 g alcohol/day	*P
	n (%)	n (%)	n (%)	
Total N	63 (57.8)	30 (27.5)	16 (14.7)	-
Moderate depression				
No	52 (55.9)	26 (28.0)	15 (16.1)	0.73
Yes	8 (61.5)	4 (30.8)	1 (7.7)	
Severe depression				
No	59 (56.7)	29 (27.9)	16 (15.4)	0.72
Yes	1 (50)	1 (50)	0 (0.0)	

ASSIST: alcohol, smoking and substance screening test. *Chi square test.

## DISCUSSION

The sociodemographic profile identified in our sample-predominantly male, over 60 years old, and married-aligns with data presented in the WHO Global Hepatitis Report 2017^16^. These characteristics are also consistent with national studies in Brazil that examined psychiatric comorbidities in HCV-infected individuals treated in specialized outpatient settings^17^ ^,^ ^18^.

Regarding gender differences, unlike previous reports that highlight higher alcohol consumption and worse hepatic profiles among men^3^ ^,^ ^9^, our study did not identify significant gender differences in alcohol use or laboratory liver parameters. This may be partially explained by the relatively low levels of alcohol consumption in our sample, which likely included fewer cases of heavy drinking. Notably, the only statistically significant gender difference observed was in depressive symptoms, which were more frequent among women - a finding that is consistent with the broader literature on gender and depression^19^.

One of the main contributions of this study is the focus on current alcohol use - including moderate patterns - among HCV-infected patients eligible for DAA treatment, a population that has been understudied. Most existing studies prioritize individuals with alcohol dependence or high daily consumption (>50 g/day)^20^, whereas our findings offer insight into patients who do not meet criteria for alcohol use disorder yet may still engage in regular drinking.

Previous work by Vieira-Castro and colleagues^21^ categorized alcohol consumption in HCV patients into low (<12 g/day), moderate (12-59 g/day), and high (>60 g/day) groups, demonstrating that moderate alcohol users showed good treatment adherence and no increased prevalence of depressive symptoms compared to lighter users. Our findings are consistent with these results: there was no significant association between alcohol use level (none, <12 g/day, ≥12 g/day) and depressive symptoms. Although we did not formally assess adherence via direct measures, all included patients were eligible for DAA therapy, and medical record review suggested satisfactory clinical follow-up.

Importantly, we found a significant association between alcohol use and tobacco use, suggesting that these patients may share behavioral risk factors that require integrated care strategies. Conversely, alcohol use was not associated with the presence of major clinical comorbidities such as hypertension or hypothyroidism, which were more frequent in the abstinent group - possibly reflecting age-related differences or previous medical advice leading to abstinence.

This study also stands out by assessing the associations between alcohol consumption, depressive symptoms, and clinical/laboratory characteristics, excluding individuals with severe psychiatric or neurological comorbidities to reduce potential confounding. This approach adds methodological rigor and allows a more targeted interpretation of findings related to moderate alcohol use.

Nonetheless, several limitations should be acknowledged. The use of retrospective medical records led to some degree of data loss and variability in clinical documentation. Additionally, the time interval between data collection and the actual initiation of DAA treatment may have introduced discrepancies in clinical status over time.

Despite these challenges, the study used validated instruments and a structured protocol, enhancing reproducibility and offering a feasible model for future research in real-world clinical settings - especially relevant in resource-limited contexts, where mental health care integration and personnel availability are ongoing challenges.

## Data Availability

The research data are presented within the article itself (available in Tables 1, 2, 3, 4, 5, and 6).
